# Communities creating change

**DOI:** 10.7554/eLife.36992

**Published:** 2018-04-09

**Authors:** Bridget M Kuehn

**Affiliations:** eLifeChicagoUnited States

**Keywords:** scientist and parent, women in science, diversity, careers in science

## Abstract

Women scientists across India are working together to build and advocate for family-friendly policies at their institutes.

India is a growing scientific powerhouse. According to statistics from the US National Science Foundation (NSF), the country published more than 100,000 articles – outpacing Japan – in 2016, and its more than 600 universities and research institutes awarded an estimated 13,000 science and engineering doctoral degrees in 2014.

Despite making up the majority of the students studying science and engineering degrees, women held only around one in eight of India’s jobs in these fields in 2005; a 2015 report found that a similar proportion of faculty positions were held by women. Many women scientists are lost in the transition to faculty positions or during their child bearing years according to Sandhya Visweswariah, who chairs the India Academy of Science Women in Science panel.

The Indian government has implemented several efforts to help retain women scientists and grow their ranks. It has created fellowship programs for women who have taken a career break. A 2017 amendment to the Maternity Benefit Act requires organizations that employ more than 10 people to permit mothers to take six months of paid maternity leave for each of their first two children. But many scientists who have children still lack support from their institutions. To change that, women scientists are working together to create onsite childcare programs, to change the way their universities account for maternity leave in tenure decisions, and to advocate for one another.

## Support systems

There is a common thread that runs through the stories of Indian women scientists, according to Nandita Jayaraj, who created the website The Life of Science with fellow journalist Aashima Dogma in 2016. Those who succeed “have a really solid support system,” says Jayaraj.

Having a supportive spouse is essential. “Society as a whole has not changed on the time scale that [government and institutional] policies are changing,” explains Vidita A. Vaidya, a professor in the department of biological sciences at the Tata Institute of Fundamental Research (TIFR) in Mumbai. “It’s a very patriarchal society. Women are the primary care givers, and women’s careers are always secondary to men’s careers.”

However, more men, especially younger men, are sharing childcare duties. When her children were very young, Maria Thaker, an assistant professor at the Indian Institute of Science’s Centre for Ecological Sciences in Bengaluru, often took them along for field studies. Now that the children are in school, she and her husband, who is also an ecologist, coordinate their work so that one stays with the children while the other heads to the field. “We feel like Superman and Clark Kent," says Thaker, “because we can’t be in same place at the same time.”

Some of Thaker’s male colleagues are also very engaged parents. Her department has adopted family-friendly policies; for example, they hold all meetings before 4:30pm to allow parents to pick up their children.

In addition, it is still common for the older generation to play an important day-to-day role in supporting families with children, according to Vaidya, who also serves on the Women in Science panel. She has her parents and in-laws nearby to help look after her child, and also has access to on-site childcare at TIFR.

But not all women are so lucky. Those who live farther from their families may face a dearth of childcare options, especially for infants. Hiring a nanny may be a financially viable option in India, but many parents prefer a childcare center, says Vaidya.

At the Indian Institute of Science Education and Research in Pune, Mayurika Lahiri, an associate professor of biology, has been instrumental in establishing an on-campus childcare center. It is so popular that it now serves 50 children of staff, faculty, and students. In 2008, when no such facility existed, Lahiri was able to arrange space on campus for a nanny to watch her infant daughter. It took six years to open an on-campus crèche. Lahiri’s department director created a childcare committee, on which Lahiri served, that interviewed daycare providers and selected the best fit. The institute provided the space and start-up funds for furniture and other materials, and now the fees paid by parents cover the day-to-day operating expenses of the facility. Lahiri shared her tips for starting a center with The Life of Science so others could follow in her footsteps.

“It gives me peace of mind,” says Lahiri, who sends her daughter to the center. “It gives you time to concentrate on your work or teaching.”

## Stopping the clock

Outdated institutional policies can also hold back women in their careers. “Our major problem in this country is having enough women in decision-making positions to be able to change attitudes and approaches,” says Visweswariah. However, this is not stopping existing senior women faculty from pushing for sensible solutions to the career challenges that mothers face in gaining tenure, landing jobs, or juggling childcare responsibilities.

Thaker sought help from Visweswariah and other biology division chairs at the Indian Institute of Science, who happened to be mostly women, after her application for tenure had a disappointing outcome. She was the first woman to be considered for tenure under a new process. The all-male panel who assessed Thaker did not factor in her six-month maternity leave, and compared her against a male colleague who had more publications. The result was that she received a three-year contract extension instead of tenure.

In response, Visweswariah and her colleagues successfully lobbied for an Institute-wide policy that provides mothers with more time to build their case for tenure without being penalized for having children. Women faculty can now ‘stop the tenure clock’ for one year per child, with a maximum of a two-year extension. For example, if a woman with two children gets tenured after seven years, her tenure and associated pay increase will be retroactively applied as if she was promoted at the end of her fifth year.

Pausing the tenure clock accounts for both the time spent on maternity leave and the time spent on infant care and nursing after returning to work. Visweswariah explains it takes some time to adjust after a child is born. “It’s not that you produce a child and then everything suddenly just disappears,” says Visweswariah. “I think male colleagues fail to understand that.”

Similar battles are taking place across India, as women try to secure places to breastfeed, promote hiring two-career couples, and support other policies that can help women stay in science. Improvements would happen faster, says Vaidya, if the government required such policies at all the institutions it funds: “These policies should not have to be reinvented every single time in every single institution all over the country.”

In the meantime, having a supportive community pushing for change is helping women stay the course. “When my colleagues in India and abroad rallied for me, I knew that I had to find a way to get past the administrative hurdles,” says Thaker. “Having a supportive community of colleagues makes all the difference.”

## Note

This Feature Article is part of the Scientist and Parent collection.

